# Automated fundus ultrasound image classification based on siamese convolutional neural networks with multi-attention

**DOI:** 10.1186/s12880-023-01047-w

**Published:** 2023-07-06

**Authors:** Jiachen Tan, Yongquan Dong, Junchi Li

**Affiliations:** 1 School of Computer Science and Technology, Jiangsu Normal University, Xuzhou , 221116 Jiangsu China; 2 Xuzhou Cloud Computing Engineering Technology Research Center, Xuzhou , 221116 Jiangsu China; 3 Xuzhou No.1 People’s Hospital, Xuzhou, 221018 Jiangsu China

**Keywords:** Fundus ultrasound images, Image classification, Deep learning, Siamese network

## Abstract

Fundus ultrasound image classification is a critical issue in the medical field. Vitreous opacity (VO) and posterior vitreous detachment (PVD) are two common eye diseases, Now, the diagnosis of these two diseases mainly relies on manual identification by doctors. This method has the disadvantages of time-consuming and manual investment, so it is very meaningful to use computer technology to assist doctors in diagnosis. This paper is the first to apply the deep learning model to VO and PVD classification tasks. Convolutional neural network (CNN) is widely used in image classification. Traditional CNN requires a large amount of training data to prevent overfitting, and it is difficult to learn the differences between two kinds of images well. In this paper, we propose an end-to-end siamese convolutional neural network with multi-attention (SVK_MA) for automatic classification of VO and PVD fundus ultrasound images. SVK_MA is a siamese-structure network in which each branch is mainly composed of pretrained VGG16 embedded with multiple attention models. Each image first is normalized, then is sent to SVK_MA to extract features from the normalized images, and finally gets the classification result. Our approach has been validated on the dataset provided by the cooperative hospital. The experimental results show that our approach achieves the accuracy of 0.940, precision of 0.941, recall of 0.940, F1 of 0.939 which are respectively increased by 2.5%, 1.9%, 3.4% and 2.5% compared with the second highest model.

## Introduction

As technology advances, people can no longer work and study without cell phones and computers today. Under high-intensity work, people’s eyes are staring at electronic screens most of the time, so more and more people suffer from eye diseases. Vitreous opacity (VO) and posterior vitreous detachment (PVD) are two common eye diseases, both of which are associated with the vitreous humor in the eye. The vitreous humor is a clear gel that contains hyaluronic acid, water (approximately 98%), and collagen fibers [1]. VO is a symptom of the blurred retina caused by opaque material within the vitreous [2]. According to epidemiological data, the onset of VO is often related to aging, and with the aging of society, there is an increase in the number of patients who have underlying diseases, such as hypertension, diabetes, and high myopia. The incidence of VO shows an increasing trend on a year-over-year basis [3]. There are many causes of VO. For example, the reaction of external light with vitamin C, oxygen and iron ions in the vitreous leads to the precipitation of water molecules [4]. Retinal hemorrhage, hypertension, and diabetes can result in vitreous liquefaction and opacity [5]. The separation of the posterior vitreous cortex from the inner retinal border membrane is known as PVD [6]. The presence of PVD is also age-dependent. Simultaneous fracture of gel liquefaction and vitreoretinal adhesions is the direct reason of PVD [7]. PVD can cause an increase in oxygen partial pressure within the vitreous [8], leading to cataracts, glaucoma, and even vision loss or blindness.

**Fig. 1:**
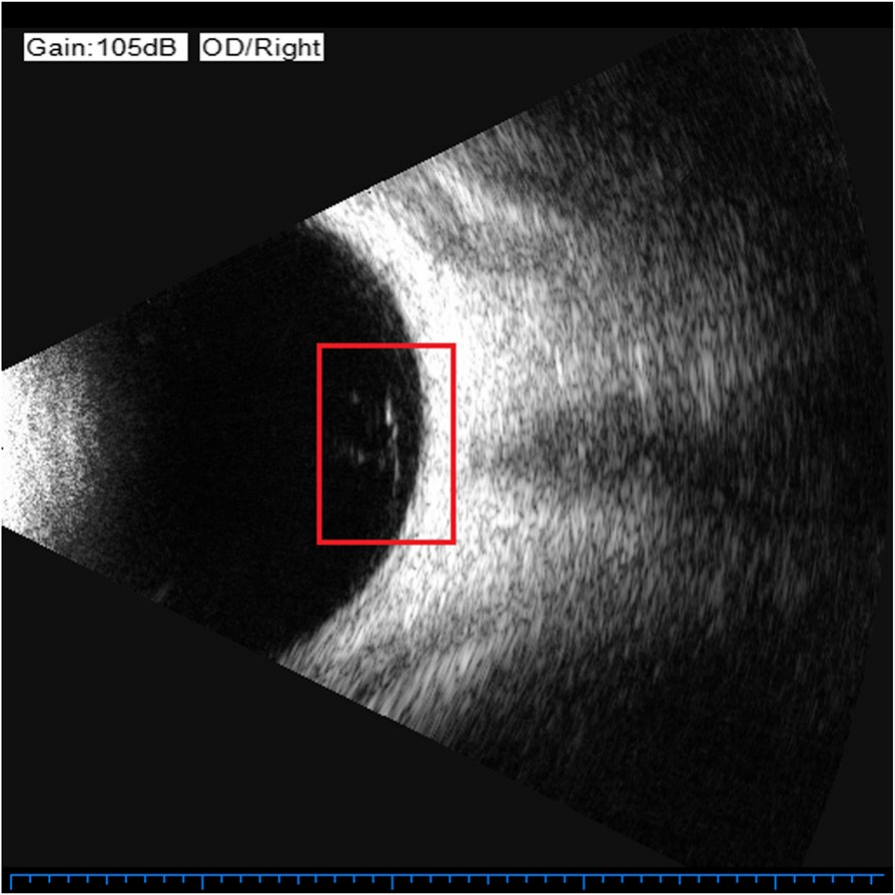
VO fundus ultrasound image

**Fig. 2:**
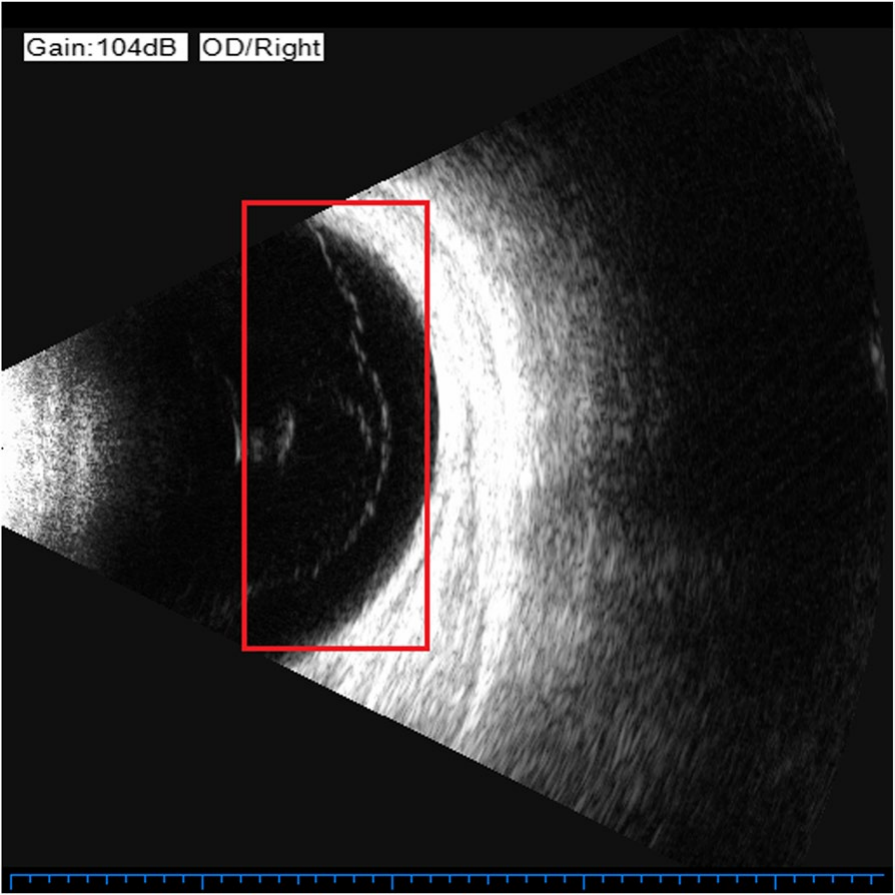
PVD fundus ultrasound image

Previously, the most common method to detect these two eye diseases was Computed Tomography (CT), but it is cumbersome, expensive, and delays in valuable treatment time for patients. Nowadays, people tend to choose more cost-effective ultrasound images to detect these two eye diseases. The ultrasound images of VO and PVD are shown in Figs. 1 and 2, respectively. The differences between VO and PVD lesions are small. As can be seen from the red rectangle in Figs. 1 and 2, most of the VO lesions are discrete white spots, while most of the PVD lesions are continuous white spots. Due to the dramatic increase in the number of patients and the shortage of doctors in recent years, some doctors have to observe thousands of ultrasound images a day, and even experienced ophthalmologists can make mistakes, so computer technology is needed to assist doctors in diagnosis.

Automatic classification of medical images has three issues. The first issue is the difficulty of extracting features for medical images, the second one is the scarcity of data. The training of neural network requires a large amount of data, but it is difficult to collect enough data due to the particularity of medical images, and the third one is that traditional convolutional neural network (CNN) is difficult to find small differences between the two kinds of images very well. For the first issue, as deep learning can automatically extract image features without manual construction, many researchers feed medical images into CNN to extract medical image features. CNN models have been successfully applied to CT image recognition of brain diseases [9], detection of lung cancer cells [10], classification of radiology imaging, cardiology imaging, and gastroenterology imaging [11]. For the second issue, Some scholars have used siamese networks and data augmentation [12] to solve the problem of data scarity. In addition, pretrained models [13] are also used to solve this problem. For the third issue, More and more researchers embed attention mechanism in their models to help them select important features [14]. Nowadays, these techniques have widely used in many fields, but there is still a gap in the VO and PVD classification task. So, in this paper, we propose an end-to-end siamese convolutional neural network with multi-attention (SVK_MA) for automatic classification of VO and PVD fundus ultrasound images. In SVK_MA, we use two branches of the pretrained VGG16 [15] model while adding two attention models (channel attention & spatial attention) to extract image features, which are used to classify the image into PVD class or VO class by K-nearest neighbor classifier (KNN). Our approach has been validated on the dataset provided by the cooperative hospital. Experimental results show that our model has an accuracy result of 0.940, precision of 0.941, recall of 0.940 and F1 of 0.939, which is better than other state-of-the-art models. As far as we know, this paper is the first time to apply deep learning model to VO and PVD classification task.

The main structure of this paper is as follows. In “Related work” section, we present the related work. In “Our method” section, we introduce our classification method, which mainly includes the framework, data preprocessing methods, SVK_MA model, attention models, classifier, and loss function. “Experiment” section mainly describes the experiments and experimental results made in this paper in detail. “Discussion” section analyzes and discusses the experimental results. The last part “Conclusion and future work” section is our conclusion and future work.

## Related work

### CNN for medical image classification

With the rapid development of computer vision, CNN has achieved good results in image processing. Medical image classification is an important part of image processing, and some scholars have applied CNN to this field. Changhun Jung proposes a shallow efficient CNN network W-Net to classify white blood cells (WBC). To advance the task, the authors investigate the applicability of transfer learning and use GAN to generate a larger dataset of WBC images which is published publicly [16]. To solve metaphase chromosome classification in cells, Abdulkadir Albayrak uses the VGG16 and Inceptionv3 networks pretrained on ImageNet for classification [17]. Chen chen applies SENet model for the first time to the classification task of liver cancer histopathological images. SENet introduces convolution-based attention module which contains channel attention and spatial attention. The results show that the attentional mechanism in SENet plays an excellent role in the histopathological images of liver cancer [14]. Junlong Cheng proposes an attention module that can capture important features in medical images from channel and spatial dimension. By stacking this module in the way of ResNet, a new ResGANet model is constructed, whose performance is 1.51–3.47 times higher than that of traditional ResNet [18]. In solving medical image segmentation problems, Junde Chen embeds channel attention and spatial attention into U-shaped networks and combines it with Inception and depth-wise separable convolution. Using this method not only reduces the parameters and complexity of the model, but also helps the model learn the inter-channel dependencies and important features in space, so as to accurately locate the diseased area and recover richer detailed features [19]. Although CNN has achieved good results in the task of medical image classification, there are still some problems. For example, CNN needs the support of a large amount of data, but it is difficult to provide sufficient data for medical images due to patient privacy and the difficulty of image annotation. Medical images often have a high resolution, which contain a lot of information, so it is difficult for CNN to select useful features. The distinctions between the different diseases in the images are so small that CNN can often misjudge them.

### Siamese network for classification

Siamese network as a kind of classical architecture has been applied to image classification. Yapin Wang uses the siamese network to classify the images of white blood cells. A small number of the same type of white blood cells and typical samples constitute a positive case pair, while other disturbances constitute a negative case pair. Since the number of positive case pairs is rare and the category is unbalanced, the author uses data amplification to increase the number of positive case pairs. Experimental results show that the siamese structure has advantages for small datasets [12]. Min Liu proposes an improved autoencoder (AE) network, which uses siamese framework and Gaussian pyramid for multi-scale processing of input images. AE network can learn effective features from breast cancer histologic images for breast cancer classification tasks [20]. Huizhen Hao proposes an image classification method of heavy mineral particles based on Siamese Adversarial network (SAN) for the first time, using the siamese network to extract the internal representation of heavy mineral particles with different polarization, and using adversarial training to reconstruct the features. The model achieves good generalization performance [21]. There are only a few annotated samples and a large number of unannotated samples in remote sensing images, which leads to the overfitting of depth models and affects the performance of scene classification. To solve these problems, Wang Miao proposes a siamese network for remote sensing image scene classification (SS-RCSN), in which Generative Adversarial network (GAN) is used to extract discriminant features from remote sensing images by unsupervised learning [22]. To sum up, siamese network is a simple and straightforward architecture that takes advantage of small datasets.

## Our method

This section first describes the framework, data preprocessing, SVK_MA model. Then, it describes the loss function and the classifier. The flow of our method is shown in Algorithm 1.

### Framework

**Fig. 3:**
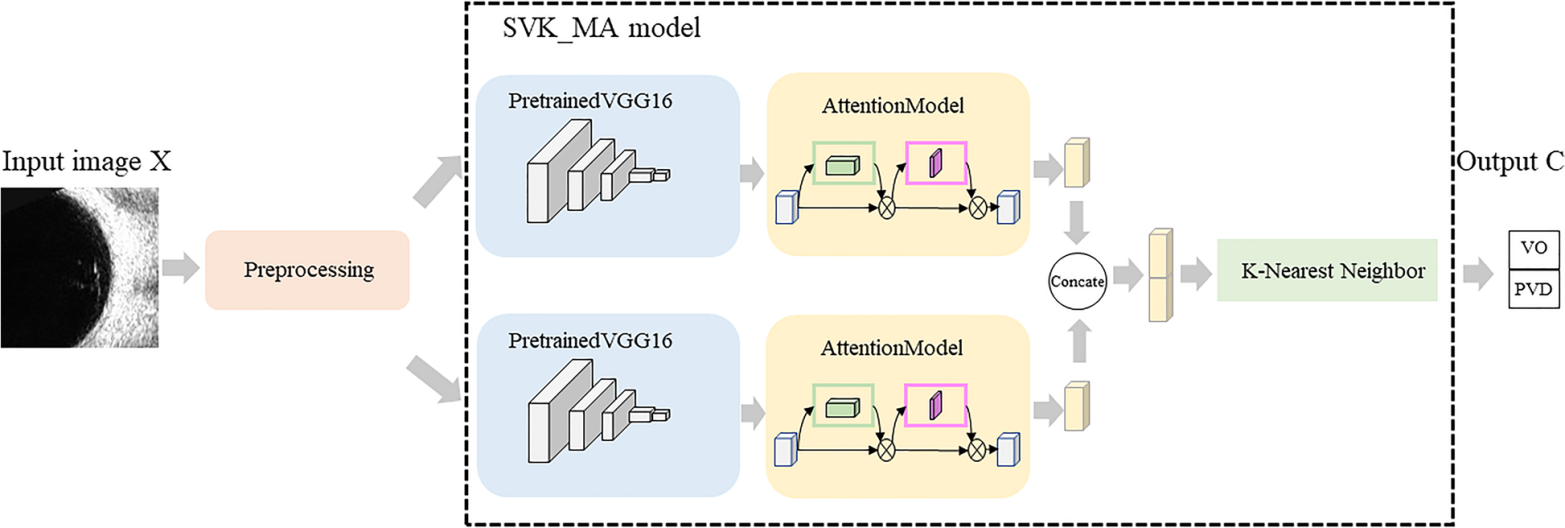
Framework of our proposed approach

Figure 3 shows the framework of our proposed approach and Algorithm 1 is the description of the framework.

**Figure:**
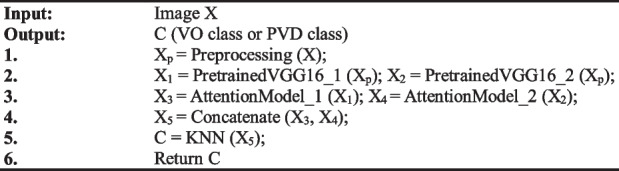
**Algorithm 1.** Our approach

In Algorithm 1, the input is an image and the output is the classification result. Line 1 represents the preprocessing of the input image, in which the normalization function is used to normalize the data to [0, 1]. In line 2, two pretrained VGG16 models with shared weights are used to extract the features of the preprocessed images. In line 3, important features are selected by using two attention models which include channel attention and spatial attention. In line 4, the two features obtained through the attention module are concatenated to get a new feature vector. Finally, in line 5, the new feature is sent into the KNN classifier to get the final classification result.

### Preprocessing

Preprocessing is an indispensable step in medical images, and the quality of the images directly affects the subsequent classification results. Data normalization and data augmentation are two common preprocessing approaches. In this section, we introduce these two approaches.

#### Data normalization

Data Normalization can reduce the degree of pixel dispersion, so as to better fit the data. In the process of model training, normalization makes the gradient more controllable and predictable, thus making the model training more stable and faster. The detailed normalization formula is shown in Eq. (1). All the normalized images are denoted as dataset D1.

1 $${xn}_{i}=\frac{{x}_{i}-{x}_{mean}}{{x}_{std}}$$

*x*_*i*_ is the $i$ th pixel point on an image of size 224 × 224 (1 ≤ $i$≤224 × 224). ${x}_{mean}$ and ${x}_{std}$ represent the mean value and the standard deviation of all pixel points. ${xn}_{i}$ denotes the value of the *i*th pixel point after normalization. Normalization is performed on each of the three RGB channels of the image. To avoid recalculating the mean and standard deviation of all pixel points before each training step, we manually set the mean and standard deviation of each channel as 0.5 before training.

#### Data augmentation

The size of the dataset has always been an important influencing factor in deep learning classification problems. A sufficiently large dataset can give a considerable improvement in classification results. However, medical images are so scarce that we can only augment the existing dataset with data augmentation methods. Two data augmentation methods are described below. One is the traditional crop, rotate, and flip data augmentation method, and the other is using DCGAN [23] to imitate real images to generate similar images.

##### Traditional approaches for data augmentation

**Fig. 4:**
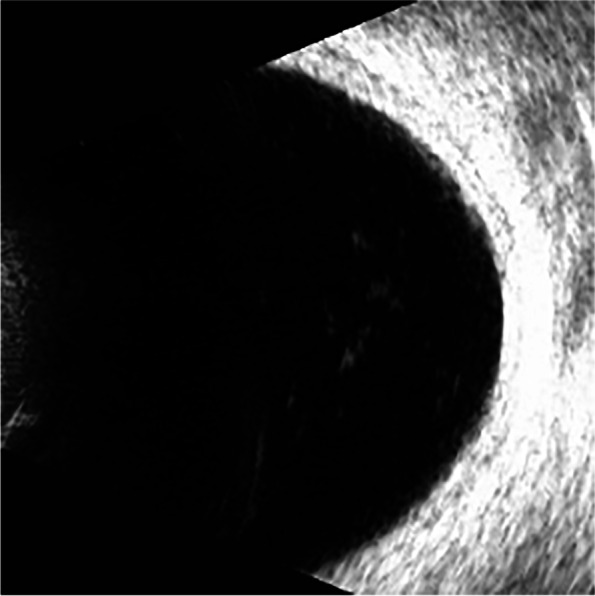
Original image

**Fig. 5:**
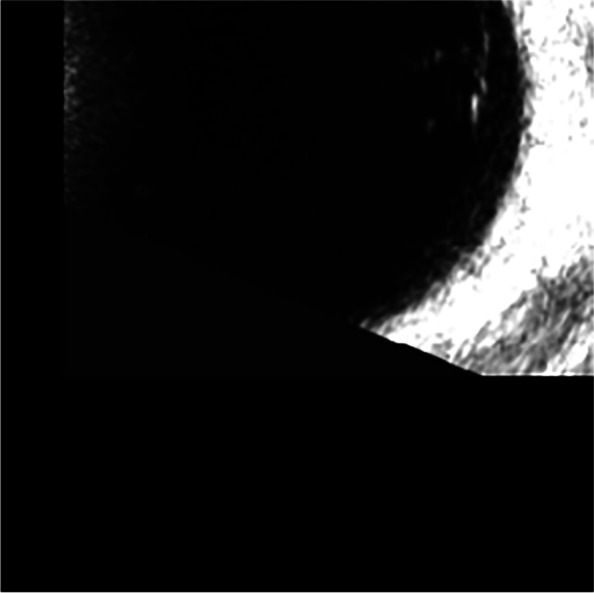
Augmented image

Images in the dataset are augmented by random cropping, up and down rotation, left and right rotation, horizontal rotation, and color jittering. The original VO image is shown in Fig. 4 and the image augmented by random clipping, rotation and color jitter is shown in Fig. 5. As can be seen in Fig. 5, part of the fundus has been cut off while part of the lesion is not shown, and the area occupied by the fundus has become smaller with the majority of the image being a useless black area. We perform traditional data augmentation on the original dataset to generate dataset D2.

##### Generative adversarial networks for data augmentation

**Fig. 6:**
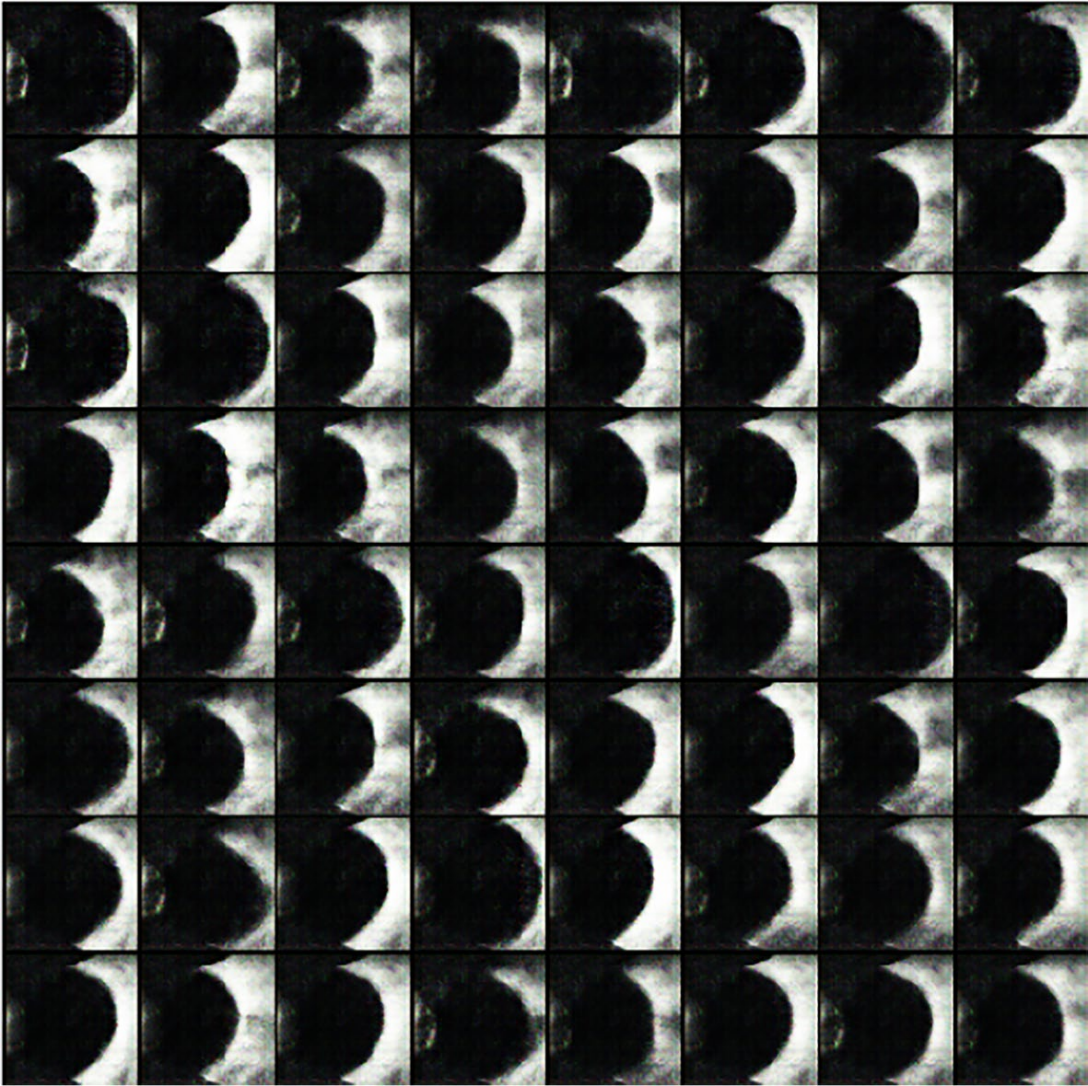
Real data

**Fig. 7:**
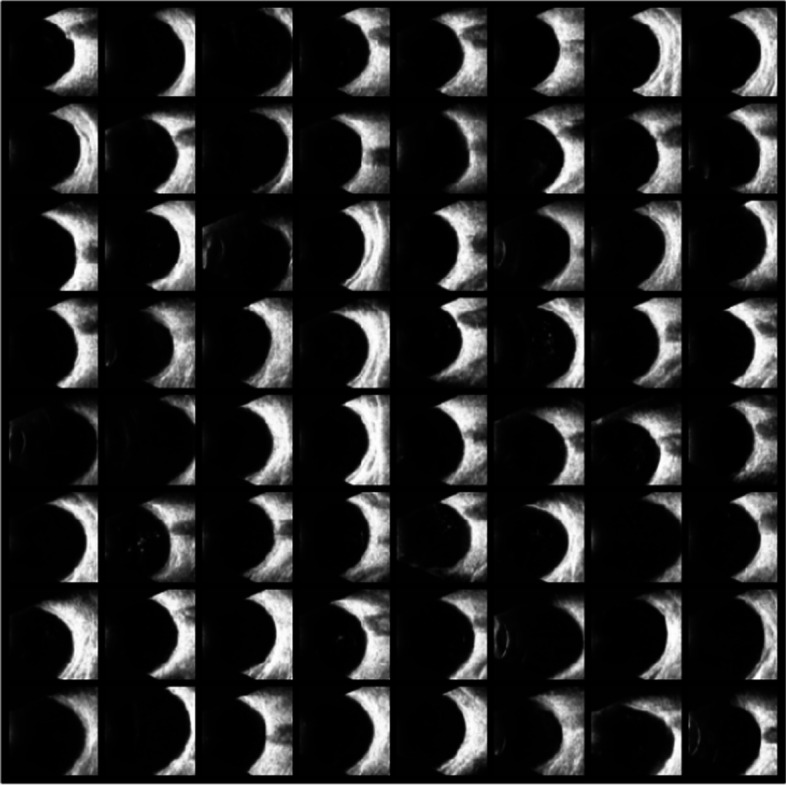
Data generated by DCGAN

Generative Adversarial Network (GAN) [24] is a way of learning through game playing and self-learning to get to the target. It generates pictures with the same distribution as the training data by learning generators and discriminators. Generators produce data as closely as possible to the distribution of the training set so that the generated data is close to the real data as much as possible. A perfect image generated by the GAN will prevent the discriminator from recognizing true or false. Deep Convolutional Generative Adversarial Networks (DCGAN) combines deep convolutional networks and GAN with certain extensions. Real data and the data generated by DCGAN are shown in Figs. 6 and 7. The images generated by DCGAN are denoted as dataset D3. The distributions of the three data sets are shown in Table 1.

**Table 1:** The distributions of three augmentation datasets

Dataset	Vitreous	Posterior Vitreous Detachment
D1	220	220
D2	249	249
D3	630	630

### Pretrained VGG16

**Fig. 8:**
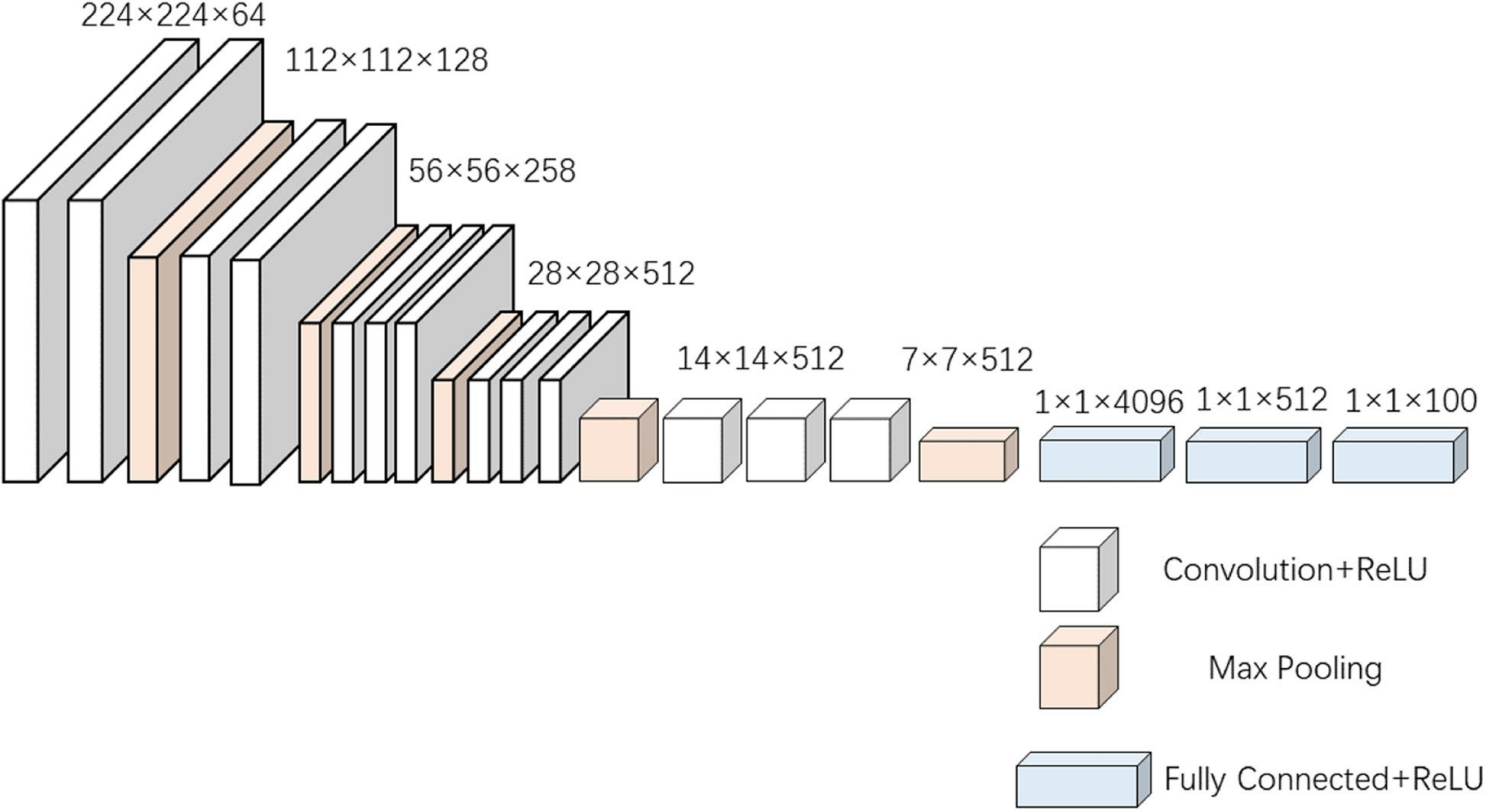
Structure of VGG16 [18]

**Fig. 9:**
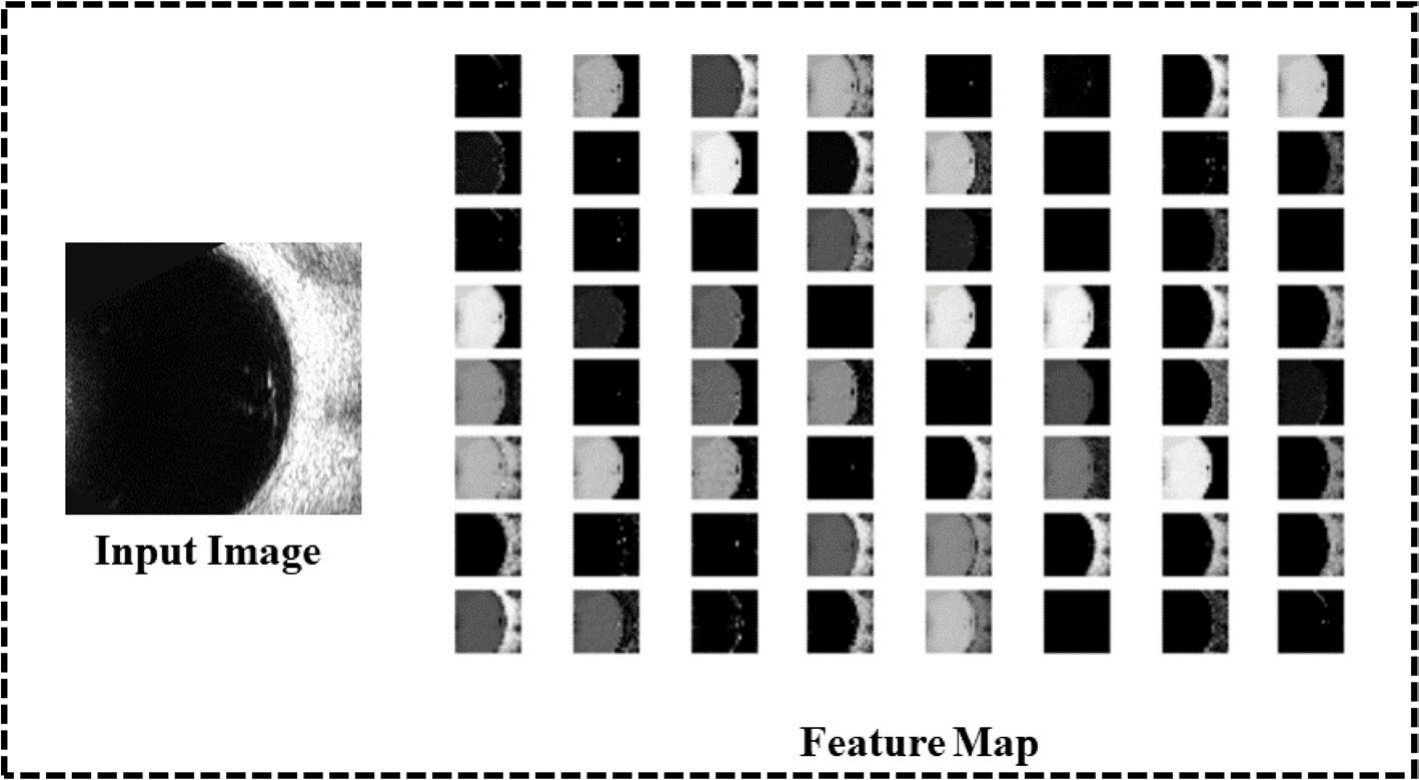
VGG16 feature map

Traditional deep convolutional neural networks (DCNN) require a large amount of labeled data for training, but it is difficult to obtain these labeled data in the medical field. So using DCNN that are pretrained on natural image datasets with lots of labels such as ImageNet has been shown to solve image classification problems [25]. The VGG16 model is a simple structured model because it is composed of a stack of convolutional and pooling layers and uses small convolutions of size 3 × 3. It also has 16 weight layers and contains a large number of parameters which has a natural advantage over other DCNNs when dealing with image problems. Therefore, we choose to use the pretrained VGG16 model. The classification ability of the network model is evaluated by using the pretrained model and transforming the weights in the network to the medical image domain. The structure of VGG16 is shown in Fig. 8 and the feature map extracted by pretrained VGG16 is shown in Fig. 9. As can be viewed from the feature map, the pretrained VGG16 model can accurately extract fundus contour features and lesion features.

### Attention model

**Fig. 10:**
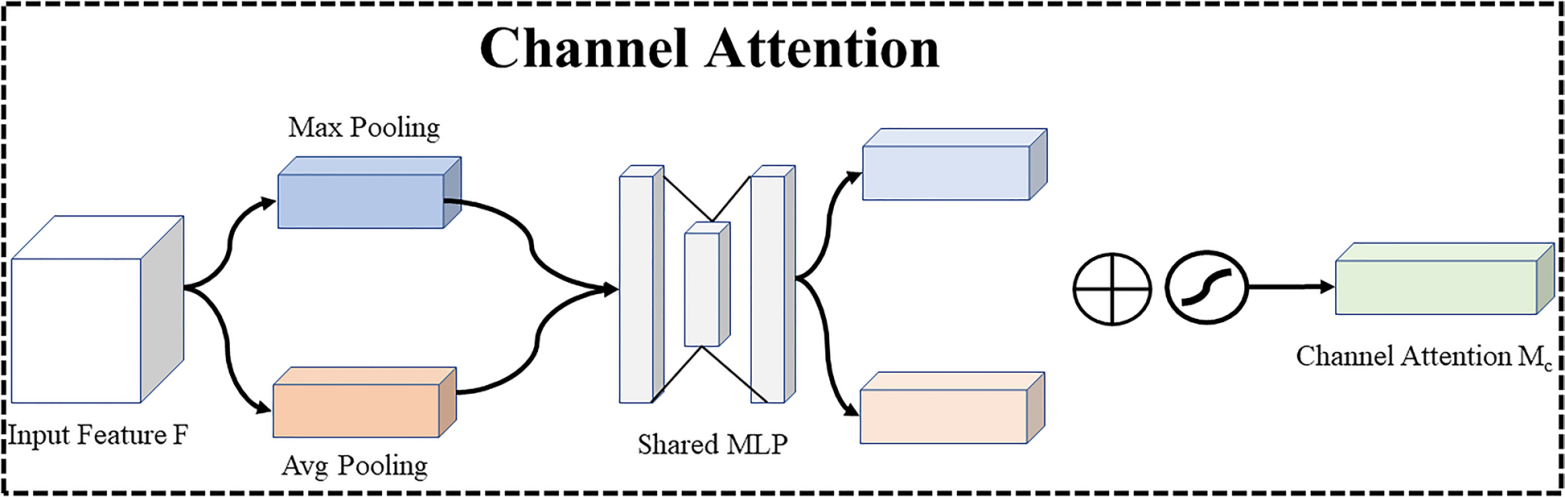
Channel Attention

**Fig. 11:**
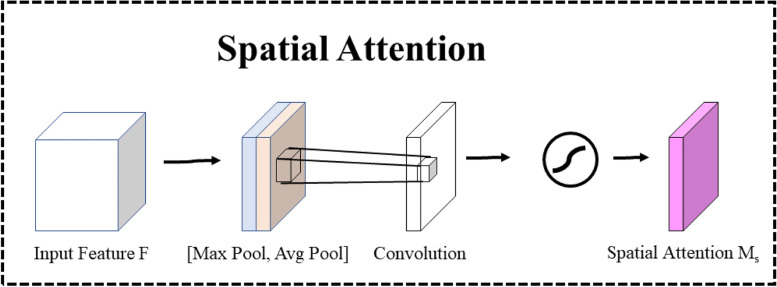
Spatial Attention

The attention model is based on the attention mechanism based on convolutions, which combines channel attention and spatial attention [26]. Given a feature, the attention model sequentially infers the attention map along two independent dimensions (channel and spatial) and then multiplies the attention map with the input feature to perform adaptive feature optimization. This attention model is lightweight and general-purpose, and its overhead is extremely small and almost negligible. The attention model can be seamlessly embedded into any CNN network. Channel attention and spatial attention are shown in Figs. 10 and 11. For channel attention, the spatial information of a feature is first aggregated by averaging and max-pooling operations, and then two different spatial context descriptors are generated. Furthermore, these two descriptors are fed forward into a network shared by both to generate the channel attention map ${M}_{c}\in {R}^{C\times 1\times 1}$, where *C* is the number of the channels. This shared network consists of a multilayer perceptron (MLP) with a hidden layer. After each spatial context descriptor is processed by the shared network, the output feature vector is fused using element-wise addition and a Sigmoid activation function. The formula for calculating channel attention is shown in Eq. (2).

2 $${M}_{c}\left(F\right)=\sigma \left(MLP\left(AvgPool\left(F\right)\right)+MLP\left(MaxPool\left(F\right)\right)\right)$$

where *F* denotes the input feature and $\sigma$ denotes the sigmoid function.

Contrary to channel attention, spatial attention focuses on areas that are information-rich, which complements channel attention. To compute spatial attention, an average pooling operation and a maximum pooling operation are firstly performed along the channel axis, and then the two are concatenated to generate an efficient feature descriptor. For the concatenated feature descriptor, a convolutional layer is used to generate a spatial attention map ${M}_{s}(F)\in {R}^{1\times H\times W}$ that encodes which regions are highlighted or suppressed. The formula for calculating spatial attention is shown in Eq. (3).

3 $${M}_{c}\left(F\right)=\sigma \left({f}^{7\times 7}(concat(AvgPool\left(F\right),Maxpool\left(F\right))\right))$$

where ${f}^{7\times 7}$ denotes a convolutional operation with a convolutional kernel size of 7 × 7 and $\sigma$ denotes the sigmoid function.

**Fig. 12:**
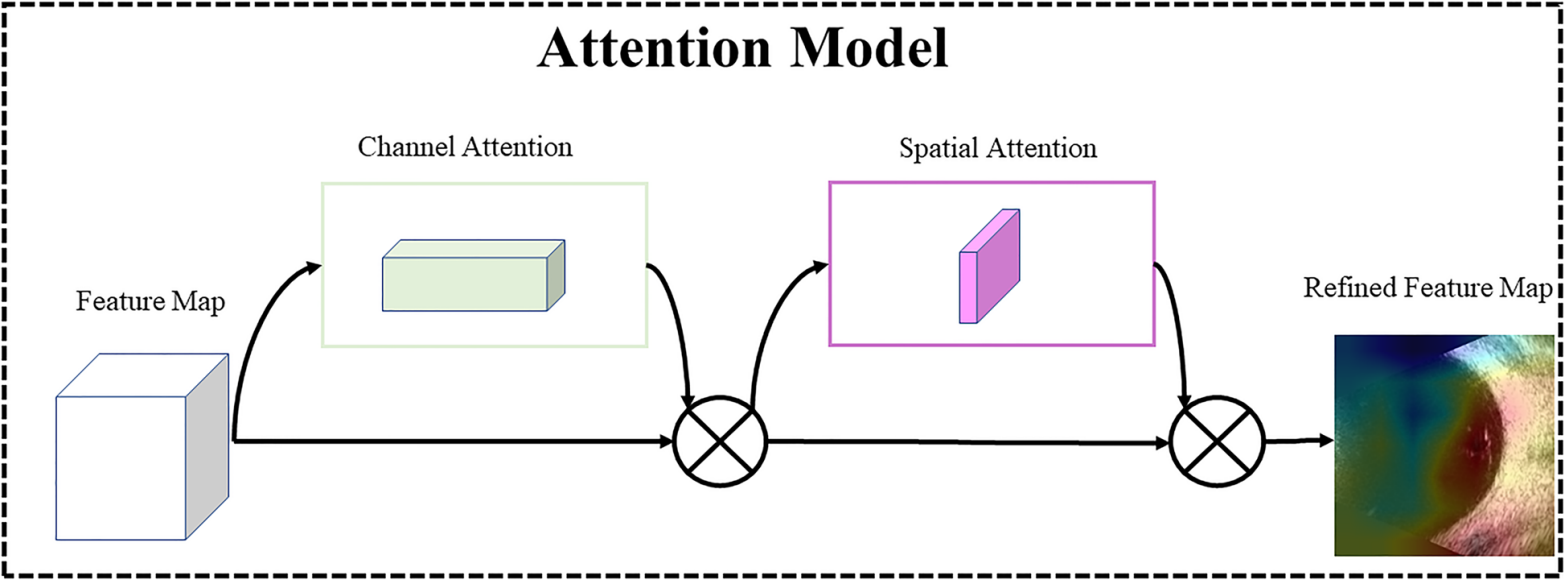
Attention model

Given an input image, two attentions (channel attention and spatial attention) are computed with complementary, focusing on the "what" and "where" respectively. The attention model consists of two attentions that are laid out sequentially. The attention model is shown in Fig. 12. As can be seen from Fig. 12, the attention model accurately focuses on the white lesion area in the fundus.

### K-Nearest neighbor classifier

K-Nearest Neighbor (KNN) Classifier is one of the most classical classifiers in machine learning [27]. For classification, the prediction of the class of a new instance is performed by majority voting based on its k nearest neighbor training instances. The KNN is described in Algorithm 2.

**Figure:**
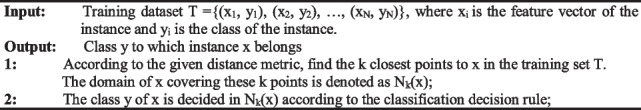
**Algorithm 2.** K-Nearest neighbor

Three basic elements of KNN are the choice of K-value, distance metric, and classification decision rule. The value of K is determined to be 2, a distance metric of Eulerian distance, and a classification rule using the majority voting method.

### Contrastive loss function

The loss function is shown in Eq. (4) [28]

4 $$L=\frac{1}{2N}{\sum }_{n=1}^{N}y{d}^{2}+(1-y)max{(margin-d,0)}^{2}$$

where d represents the Euclidean distance between the two sample features, y is the label of whether the two samples match, y = 1 means the two samples are match, y = 0 means no match, the margin is the threshold.

This loss function is mainly used in dimensionality reduction, where two originally similar samples are still similar in the feature space after feature extraction, while two samples that are originally dissimilar are still dissimilar.

Due to the use of the contrastive loss function, two images are randomly selected from the dataset and fed into the model as a pair during training. If the two images are in the same class, we call it a positive image pair and set it to 1. If the two images are not in the same class, we call it a negative image pair and set it to 0. The value of the loss function can be well expressed as the matching degree of the input samples and can be well used to train the model for extracting features. When y = 1 (samples are similar), only the first half of the loss function remains, and if the Euclidean distance between the two samples is too large at this point, it indicates that the model is currently poorly classified, so the loss is increased. When y = 0 (samples are not similar), only the second half of the loss function remains, and if the Euclidean distance of the dissimilar images becomes smaller, its loss becomes larger.

## Experiment

### Dataset

**Fig. 13:**
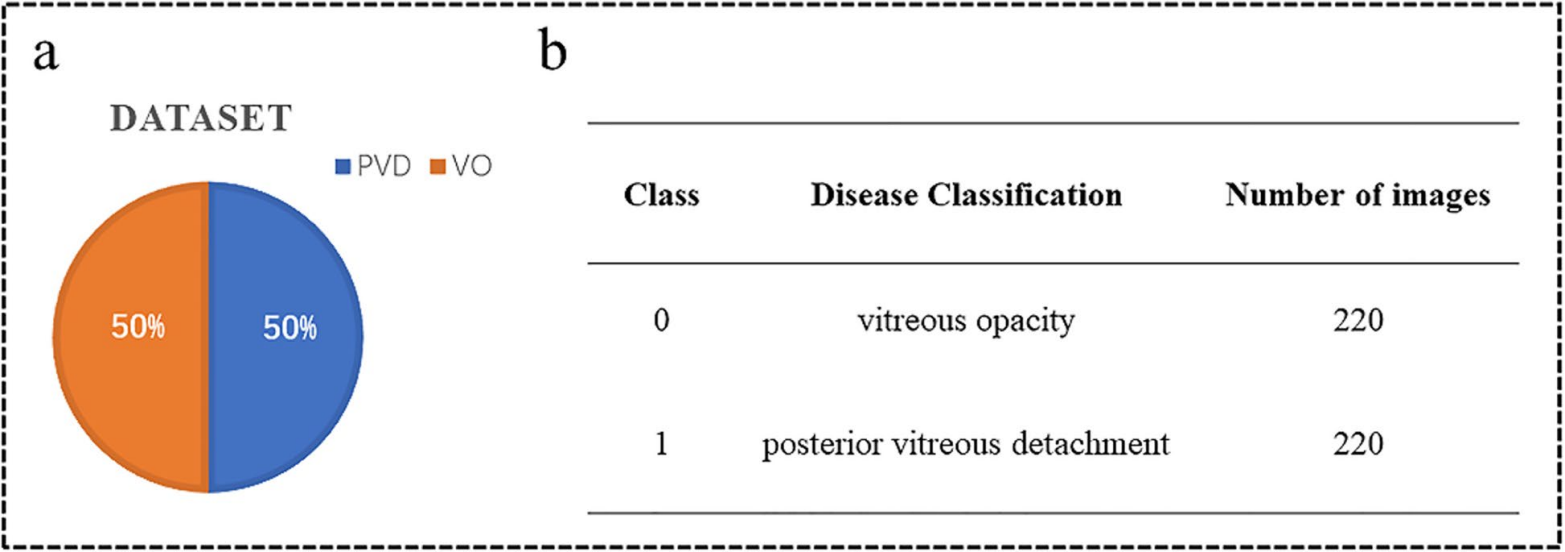
**a** indicates the percentage of PVD and VO and **b** shows the number of both diseases

**Fig. 14:**
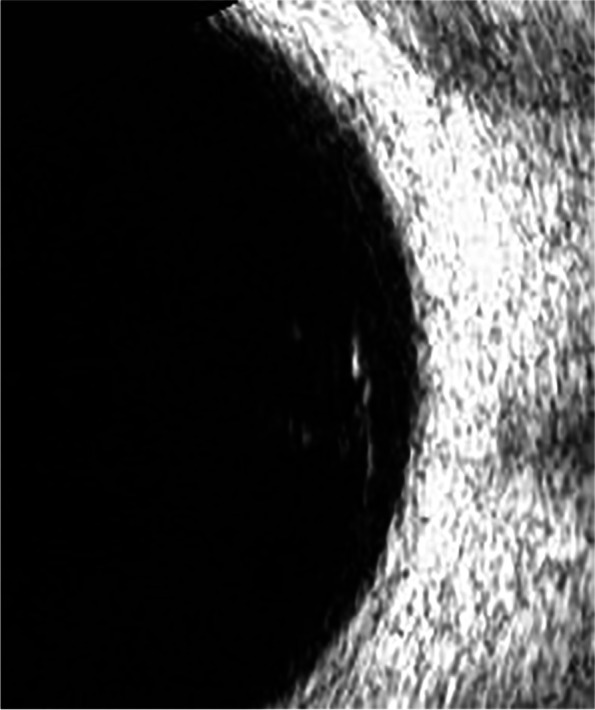
VO cropped image

**Fig. 15:**
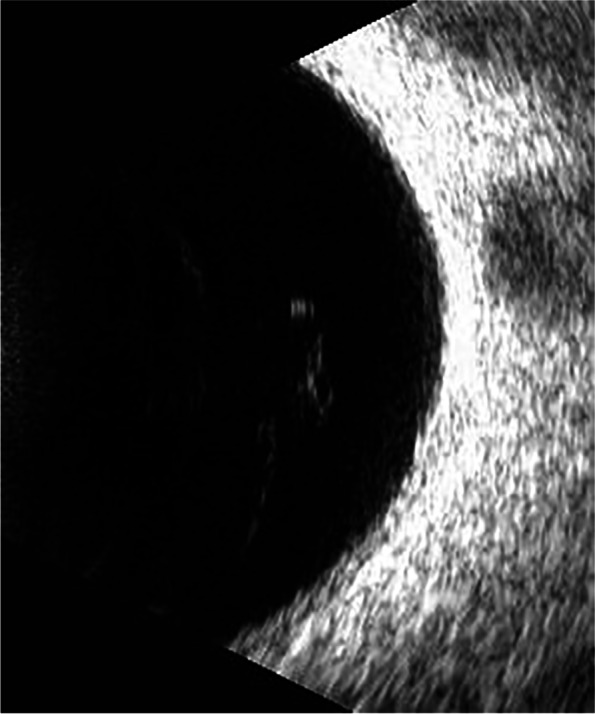
PVD cropped image

PVD and VO fundus ultrasound images are acquired from the Xuzhou No.1 People’s Hospital by desensitization. Doctors use the ultrasound instrument to scan the patients’ right and left eyes at random to produce different angles of the fundus ultrasound image. We classify the images collected by doctors according to categories, and the images with the same category are stored in the same folder. The distribution of the dataset is shown in Fig. 13. The dataset contains a total of 440 fundus ultrasound images, with a 50% split between PVD and VO to ensure balanced categories. Since the images we collected are all taken with the same device, the relative positions of the fundus in all images barely changed. According to the principle of maximizing the preservation of fundus and reducing the useless area, the rectangle part of the image is selected whose upper left corner is located at (100,300) and lower right corner is located at (400,50). This is done automatically by the program. The PVD and VO cropped images are shown in Figs. 14 and 15, respectively.

### Evaluation metrics

To measure the classification effectiveness, in this experiment we use four evaluation metrics: Accuracy, Precision, Recall, and F1, which are defined by the Eqs. 5, 6, 7 and 8.

5 $$Accuracy=\frac{TN+TP}{TP+FP+TN+FN}$$

6 $$Precision=\frac{TP}{TP+FP}$$

7 $$Recall=\frac{TP}{TP+FN}$$

8 $$F1=\frac{2\times Precision\times Recall}{Precision+Recall}$$

where TP denotes the number of positive class images classified as positive class, FN denotes the number of positive class images classified as negative class, FP denotes the number of negative class images classified as positive class, and TN denotes the number of negative class images classified as negative classes.

### Experiment setup

The environment used for this experiment is PyTorch 1.0.0 and the system environment is Windows 10. The experiments are conducted using an NVIDIA Telsa T4 GPU. The detailed experimental parameters are shown in Table 2.

**Table 2:** Experimental parameters

Parameters	Values
Learning rate	0.00001
Optimizer	Adam
Batch Size	50
Epoch	50

For datasets D1 and D2, we divided the training and test datasets in a ratio of 7:3. 70% of the data are used to train and 30% to test the classification performance. For the dataset D3 generated by DCGAN, we use all the generated images for training and all the original images as the test dataset.

### Experiment results

#### Dataset experiment

In this section, we first test different datasets to analyze the impact of datasets generated by different data augmentation methods on the classification performance. We use the SVK_MA to perform experiments on D1, D2, D3 datasets individually. The results of different datasets are shown in Table 3.

**Table 3:** The results of the experiments using SVK_MA on different datasets

Dataset	Accuracy	Precision	Recall	F1
D1	**0.940**	**0.941**	**0.940**	**0.939**
D2	0.870	0.876	0.868	0.868
D3	0.609	0.760	0.610	0.544

As seen in Table 3, D1 achieves optimal performance on all metrics. D1 is 8% higher than D2 and 54% higher than D3 in the metric of accuracy. Data augmentation can improve classification performance for most models, but due to the specific nature of the medical images used in this paper, traditional methods such as rotation, cropping, color shifting, and recent data augmentation methods of.

DCGAN fail to improve classification results. Based on the above experimental results, we use the D1 dataset for the following experiments.

#### CNN branch experiment

Siamese network has two branching networks with shared weights. These two networks use convolutional layers to extract features from the input images. To obtain high-quality image features, we replace the branch network with various pretrained models and compare the results between the different pretrained models. The structure and parameters of the siamese networks with different branch networks are shown in Table 4. The experimental results for the different branches of the model on dataset D1 are shown in Table 5. SVK_MA achieved the best results, with a 10.3% and 3.8% improvement in accuracy over SAK_MA and SRK_MA, respectively.

**Table 4:** Structure and parameters of different branch networks

VGG16 branch(SVK_MA)	Alexnet branch(SAK_MA)	Resnet18 branch(SRK_MA)
Conv, 64, 3 × 3	Conv, 64, 11 × 11	Conv, 64, 7 × 7
Conv, 64, 3 × 3	Maxpool, 3 × 3	BatchNorm, 64
Maxpool, 2 × 2	Conv, 192, 5 × 5	Maxpool, 3 × 3
Conv, 128, 3 × 3	Maxpool, 3 × 3	Conv, 64, 3 × 3
Conv, 128, 3 × 3	Conv, 384, 3 × 3	BatchNorm, 64
Maxpool, 2 × 2	Conv, 256, 3 × 3	Conv, 64, 3 × 3
Conv, 256, 3 × 3	Conv, 256, 3 × 3	BatchNorm, 64
Conv, 256, 3 × 3	Maxpool, 3 × 3	Conv, 64, 3 × 3
Conv, 256, 3 × 3	**Attention Model**	BatchNorm, 64
Maxpool, 2 × 2	FC(4096)	Conv, 64, 3 × 3
Conv, 512, 3 × 3	FC(512)	BatchNorm, 64
Conv, 512, 3 × 3	FC(100)	Conv, 128, 3 × 3
Conv, 512, 3 × 3		BatchNorm, 128
Maxpool, 2 × 2		Conv, 128, 3 × 3
Conv, 512, 3 × 3		BatchNorm, 128
Conv, 512, 3 × 3		(downsample) Conv, 128, 1 × 1
Conv, 512, 3 × 3		BatchNorm, 128
Maxpool, 2 × 2		Conv, 128, 3 × 3
**Attention Model**		BatchNorm, 128
FC(4096)		Conv, 128, 3 × 3
FC(512)		BatchNorm, 128
FC(100)		Conv, 256, 3 × 3
		BatchNorm, 256
		Conv, 256, 3 × 3
		BatchNorm, 256
		(downsample) Conv, 256, 1 × 1
		BatchNorm, 256
		Conv, 256, 3 × 3
		BatchNorm, 256
		Conv, 256, 3 × 3
		BatchNorm, 256
		Conv, 512, 3 × 3
		BatchNorm, 512
		Conv, 512, 3 × 3
		BatchNorm, 512
		(downsample) Conv, 512, 1 × 1
		BatchNorm, 512
		Conv, 512, 3 × 3
		BatchNorm, 512
		Conv, 512, 3 × 3
		BatchNorm, 512
		**Attention Model**
		FC(512)
		FC(100)

**Table 5:** The results of different branching models

Model	Accuracy	Precision	Recall	F1
SVK_MA	**0.940**	**0.941**	**0.940**	**0.939**
SAK_MA	0.852	0.852	0.822	0.852
SRK_MA	0.905	0.908	0.906	0.906

#### Attention model Ablation experiment

**Fig. 16:**
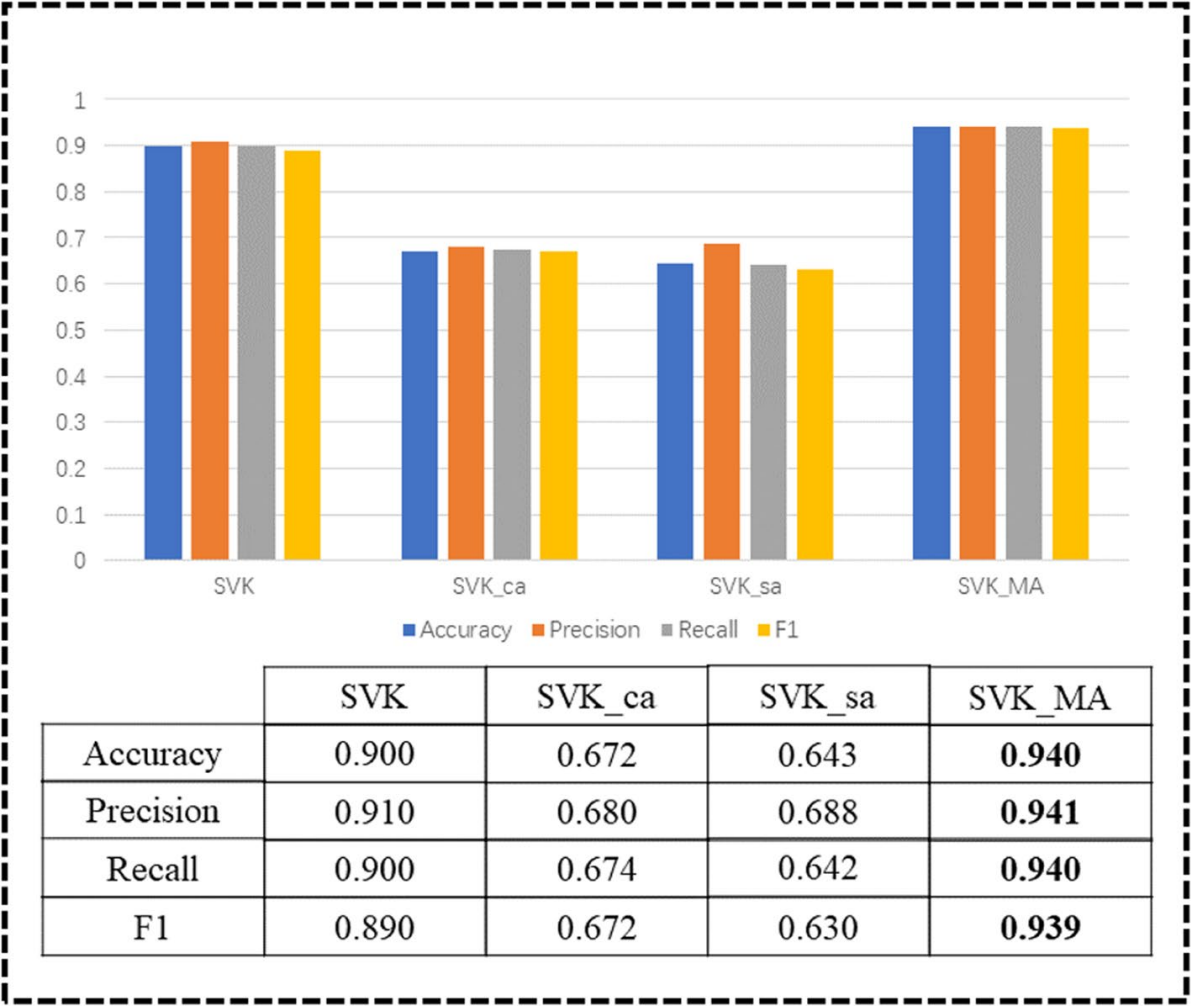
Ablation experiments results

**Fig. 17:**
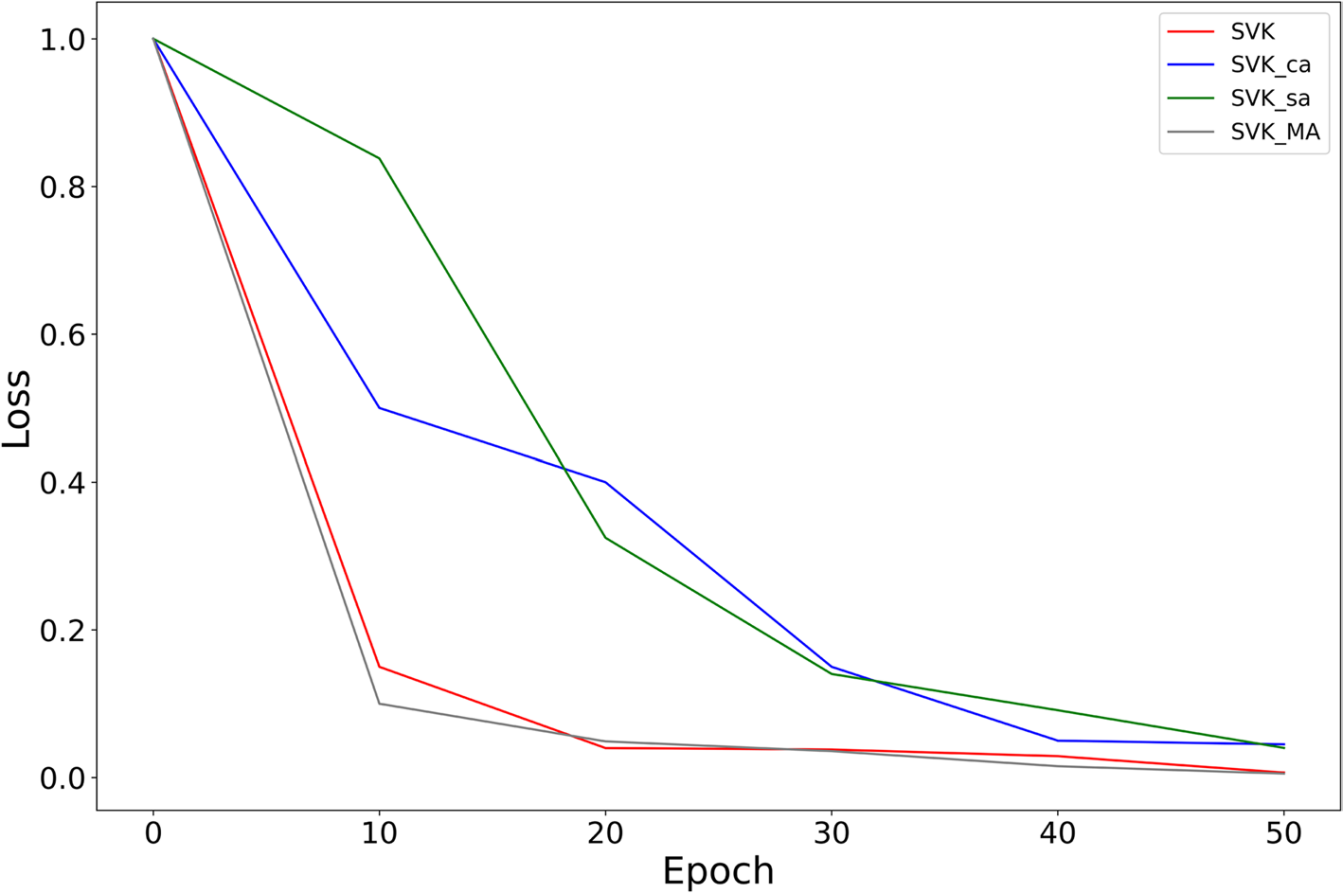
Variation in the loss for different models during training

For the validation of the effectiveness of channel attention and spatial attention of the model, we conduct ablation experiments for each attention. We compare four models, without attention (SVK), with channel attention (SVK_ca), with spatial attention (SVK_sa) and SVK_MA. The results of the experiment are shown in Fig. 16 and the losses when training the different networks are shown in Fig. 17. From the experimental results, we can see that the model is much more effective when it has both attention mechanisms than just a single one. Moreover, we can see from Fig. 17 that the loss of SVK_MA converge fastest in the10th epoch.

#### Parameter experiments

In order to find the optimal K value, we conduct parameter experiments on the K value in KNN, and the specific experimental results are shown in Table 6. When the value of k is 2,4,6,8 and 10, the result is the best. In order to reduce the parameters, we choose the 2.

**Table 6:** The results of different K value

K	Accuracy	Precision	Recall	F1
2	**0.940**	**0.941**	**0.940**	**0.939**
4	**0.940**	**0.941**	**0.940**	**0.939**
8	**0.940**	**0.941**	**0.940**	**0.939**
10	**0.940**	**0.941**	**0.940**	**0.939**
20	0.918	0.920	0.920	0.920
40	0.918	0.920	0.920	0.920
80	0.918	0.920	0.920	0.920
100	0.918	0.920	0.920	0.920
200	0.918	0.920	0.920	0.920

#### Classifier experiment

Due to the differences in principles and mechanisms between different classifiers, we have done experiments on different classifiers and selected the classifier with the best performance. The results of different classifiers on dataset D1 are shown in Table 7. KNN achieved the highest result, and compared with SVM, RandomForest and GBDT, the accuracy was improved by 106%, 5.1% and 15.7% respectively.

**Table 7:** The results of different classifiers

Classifiers	Accuracy	Precision	Recall	F1
KNN	**0.940**	**0.941**	**0.940**	**0.939**
SVM	0.456	0.264	0.456	0.310
RandomForest	0.894	0.898	0.884	0.894
GBDT	0.812	0.818	0.812	0.814

#### Comparative experiment

In addition to comparing the components of the model, we also compare it with existing baseline models(VIT, Swin-T, VGG16, Alexnet and Resnet18), and the results are shown in Table 8. SVK_MA achieves the accuracy of 0.940, precision of 0.941, recall of 0.940, F1 of 0.939 which are respectively increased by 2.5%, 1.9%, 3.4% and 2.5% compared with the second highest model.

**Table 8:** The results of comparison with classical models

Model	Accuracy	Precision	Recall	F1
SVK_MA	**0.940**	**0.941**	**0.940**	**0.939**
VIT [29]	0.917	0.923	0.909	0.916
Swin-T [30]	0.880	0.923	0.845	0.882
VGG16	0.903	0.906	0.903	0.903
Alexnet [31]	0.841	0.831	0.851	0.839
Resnet18 [32]	0.876	0.876	0.876	0.876

## Discussion

In this section, we focus on several aspects of SVK_MA in terms of its ability to classify PVD and VO.

### Normalization vs. Data augmentation

The size of the dataset has been considered an important influence on the classification result in the field of medical images. Medical image data is very scarce. It is difficult to collect enough data for the model to learn, which poses a huge challenge for the classification task. To solve this problem, generative network models such as DCGAN are widely used in the field of deep learning to solve the problem of lack of data. DCGAN imitates the original data to generate similar data to augment the dataset, but the generated images may not distributed in the same way as the original images. In addition to using DCGAN, we also use traditional methods such as flipping, cropping, and rotating to augment the dataset. Traditional methods can maintain the same distribution as the original image, but since the original image is not symmetrical, these transformations often destroy the lesion area, leading to the model failing to recognize and classify accurately. It can also be seen from Table 3 that a simple normalization of the original dataset works best.

### VGG16 branch vs. Other CNN branches

We compare the model classification effect with our original method of using VGG16 as a branch by replacing the two branches of the siamese network with Alexnet and Resnet18. Alexnet reduces the size of the features by resizing the convolution kernel and using overlapping max pool layers to fully extract the image features. However, the shallow layers of the Alexnet network make it difficult to fully represent image features. The Alexnet also has a large number of parameters and can be easily overfitted. VGG16 compensates for the shortcomings of Alexnet by increasing the depth of the network and decreasing the size of the convolutional kernel and pooling layers. Resnet18 adds network depth, shortcut, and residual blocks to the vgg16 prototype, reducing the amount of calculation and solving the overfitting problem caused by networks with too many layers. However, due to the unique nature of PVD and VO images, the Resnet18 is often susceptible to overfitting when dealing with such images with little data and simple features. From Table 5, we can see that the best classification results are achieved when using VGG16 as a branch of the siamese network.

### Multi-attention vs. Single attention

Channel attention focuses on the channel dimension and can identify more representative feature, but it cannot identify the important parts of a feature. Spatial attention just compensates for the shortcomings of channel attention by focusing on the parts of the feature that contain valid information. We use the two attention mechanisms separately in the model alone, in contrast to the sequential combination of the two attention mechanisms. From Fig. 4, we can see that the best result of the model is achieved by including both channel attention and spatial attention. It may be because adding single channel attention or spatial attention to the model may make the model pay too much attention to the channel dimension or spatial dimension, while ignoring the information of other dimensions, resulting in a lower result than the case without attention. Adding both channel and spatial attention to the model allows all dimensions to be focused, so the best results can be achieved.

### KNN vs. Other classifiers

KNN is one of the most classic machine learning classification methods. SVM is robust but takes a long time to process large training samples. GBDT is suitable for low-dimensional data and increases the computational complexity significantly when dealing with high-dimensional data. RandomForest can resist overfitting but is slow to train. GaussionNB is fast but inflexible and has poor classification results. KNN is faster than SVM, GBDT, and RandomForest, more accurate than GaussionNB and less sensitive to anomalous samples. It can also be seen from Table 7 that using KNN is the best result.

### SVK_MA vs. Other models

**Fig. 18:**
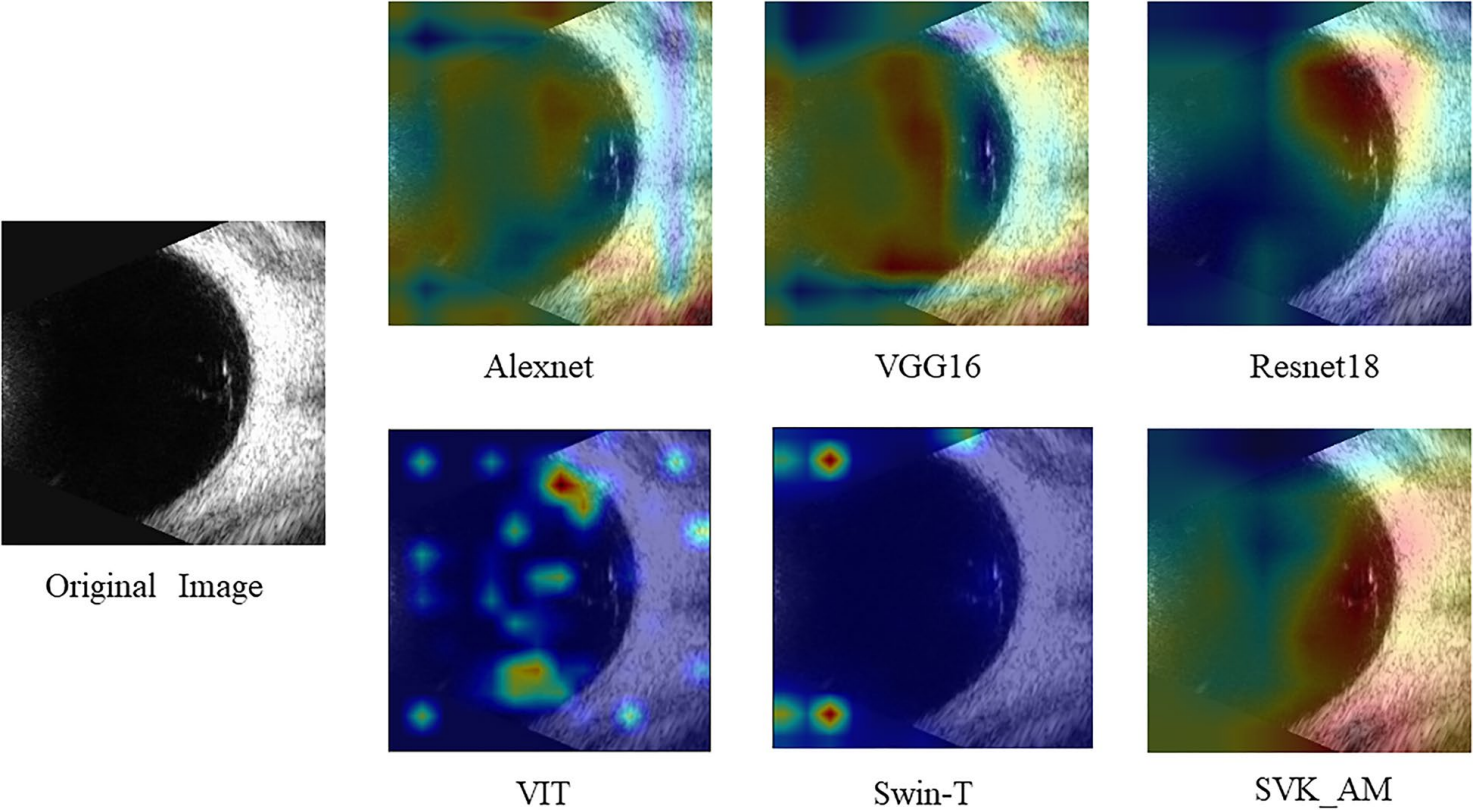
Comparison of heat maps between different models

We propose SVK_MA model is a network based on Siamese network architecture. Due to the characteristics of the siamese network, SVK_MA requires only a few training data can achieve high accuracy and has higher stability for category imbalance. It can be seen from Fig. 18 that Alexnet’s focus area is very scattered and not fully focus on the eyeball and lesion. Compared with Alexnet, the focus area of VGG16 is more concentrated, but it is mainly concentrate on the eyeball, and there is almost no focus on the lesion. Compared with the first two, Resnet18 focuses on both the eyeball and the lesion, but it only focuses on the part of the lesion and does not concentrate on all the lesions and the fundus. SVK_MA accurately focuses on all lesions and fundus and accurately finds out the most helpful and informative area for classification results. Although the two Transformer models, VIT and Swin-T, can exceed some traditional CNN models, from the attention map we can find that the concerns of both models are irrelevant areas. This may be because Transformer models are built by self-attention, with a large number of model parameters, so a large amount of data is needed for training. Due to the limited size of the dataset in this experiment, some deviated preferences are generated in the model training. As can be seen from Table 8, SVK_ MA is superior to other classical CNN models in various data.

## Conclusions and future work

In this work, we collect fundus ultrasound images of PVD and VO from real hospitals and propose a CNN model SVK_MA to assist doctors in distinguishing between the two diseases. SVK_MA can automatically obtain the features of fundus ultrasound images and automatically classify diseases according to the features. It is an end-to-end model without manual distinction by doctors. This paper introduces the process of data augmentation, the construction of the network model and the test experiment of each component of the network in detail. Finally, SVK_MA is tested on the real images, and the accuracy, precision, recall, and F1 reach 0.940, 0.941, 0.940, and 0.939, respectively.

In the future, we plan to improve our work in the following aspects. First, we need to collect more fundus ultrasound images to enrich the feature information of the model. Secondly, we will develop models with larger scale and better generalization performance, which can apply the existing models to other fields. Last, we will focus on incorporating more information related to eye diseases, such as the shape of the blood vessels in the eye, detection of the lesion area, and more cases of patients, to improve the generalizability of our approach.

## Data Availability

The dataset is available only upon request by emailing Yongquan Dong (tomdyq@163.com).
